# Emerging topics in *C. elegans* aging research: Transcriptional regulation, stress response and epigenetics

**DOI:** 10.1016/j.mad.2018.08.001

**Published:** 2018-08-19

**Authors:** Martin S. Denzel, Louis R. Lapierre, Hildegard I.D. Mack

**Affiliations:** a Max Planck Institute for Biology of Ageing, Cologne, Germany; b Department of Molecular Biology, Cell Biology and Biochemistry, Brown University, Providence, RI, USA; c University of Innsbruck, Innsbruck, Austria

**Keywords:** *C. elegans*, Aging, Proteostasis, Epigenetic, Transcription factors

## Abstract

Key discoveries in aging research have been made possible with the use of model organisms. *Caenorhabditis elegans* is a short-lived nematode that has become a well-established system to study aging. The practicality and powerful genetic manipulations associated with this metazoan have revolutionized our ability to understand how organisms age. 25 years after the publication of the discovery of the *daf-2* gene as a genetic modifier of lifespan, *C. elegans* remains as relevant as ever in the quest to understand the process of aging. Nematode aging research has proven useful in identifying transcriptional regulators, small molecule signals, cellular mechanisms, epigenetic modifications associated with stress resistance and longevity, and lifespan-extending compounds. Here, we review recent discoveries and selected topics that have emerged in aging research using this incredible little worm.

## 1. Longevity-associated transcriptional regulators

The model organism *C. elegans* was fundamental in establishing that aging is regulated by cellular signaling pathways that sense environmental or internal stress (Kenyon, 2010). Examples for such stresses or perturbations that affect *C. elegans* lifespan include reduced insulin/IGF-1 like signaling (IIS), germline ablation, dietary restriction (DR, i.e. reduced food intake without starvation), reduced TOR-activity, and inhibition of the mitochondrial electron transport chain (ETC) (Kenyon, 2010; Riera et al., 2016). Yet, it is increasingly becoming clear that different upstream stimuli employ partially overlapping sets of downstream mediators and processes that ultimately produce lifespan extension. Examples for such mediators include the widely conserved transcription factors DAF-16 (FOXO), HLH-30 (TFEB), PHA-4 (FOXA), HIF-1 (HIF1A), HSF-1 (HSF1) SKN-1 (NRF2), as well as nuclear hormone receptors (Table 1). Notably, maintaining coordinated expression of genes in various stress resistance pathways and avoiding transcriptional drift allows animals to live longer (Rangaraju et al., 2015). Currently, the regulation and integration of the activity of these transcription factors with environmental and metabolic stimuli is not completely understood. Recent studies have focused on characterizing modifications and interactions between these transcription factors and new regulators with roles in lifespan modulation.

### 1.1. DAF-16 (FOXO)

The sole *C. elegans* member of the evolutionarily conserved forkhead box O (FOXO) family of transcription factors is encoded by the gene *daf-16*, which plays key roles in maintaining homeostasis under stress and in extending lifespan in response to various stimuli (reviewed in (Eijkelenboom and Burgering, 2013; Kenyon, 2010)). How do cells modulate DAF-16 activity? Subcellular localization, transcriptional activity and stability of DAF-16 are tightly regulated by posttranslational modifications (Calnan and Brunet, 2008). When activity of the insulin/IGF1-like receptor is reduced, phosphorylation of DAF-16 by AKT-1/2 and binding of 14–3-3 proteins ceases and DAF-16 can accumulate in the nucleus (Kenyon, 2010; Manning and Toker, 2017). The nuclear import of DAF-16 can also be modulated by reactive oxygen species (ROS) via disulfide bond formation with transportin-1 (IMB-2) (Putker et al., 2013). Several factors have been identified that regulate DAF-16 by targeting its upstream kinase AKT-1, such as the SCF ubiquitin ligase complex and *daf-12* (cf. below) via micro-RNAs *mir-84* and *mir-243* (Chaudhari and Kipreos, 2017; Shen et al., 2012). Post-translational modifications of DAF-16 include phosphorylation by AAK-2 (AMPK) (Greer et al., 2007), acetylation by CBP-1 (p300/CBP) (Chiang et al., 2012b), methylation by PRMT-1 (PRMT1) (Takahashi et al., 2011), ubiquitylation by RLE-1 (RC3H1/Roquin-1) (Li et al., 2007) and deubiquitylation by MATH-33 (USP7/HAUSP) (Heimbucher et al., 2015).

MBK-1, the *C. elegans* ortholog of the mammalian FOXO1 kinase DYRK1A also modulates DAF-16, but a kinase-substrate relationship has not formally been established (Mack et al., 2017). Nuclear factors also modulate DAF-16 function and they include histone deacetylase SIR-2.1 (Berdichevsky et al., 2006), transcriptional regulator HCF-1 (HCF1) (Li et al., 2008), nuclear mRNA exporter HEL-1 (Seo et al., 2015), the SWI/SNF-complex (Riedel et al., 2013) and, potentially, ZFP-1 (AF10) (Riedel et al., 2013; Singh et al., 2016). In addition, the plasma membrane-associated protein EAK-7 (TLDC1) (Alam et al., 2010), the PP4 regulatory subunit SMK-1 (PPP4R3A) (Wolff et al., 2006), the neuronal micro-RNA *mir-71* (Boulias and Horvitz, 2012), the cytoskeletal adapter protein KRI-1 (KRIT1/CCM1) (Berman and Kenyon, 2006), the transcription elongation factor TCER-1 (TCERG1) (Ghazi et al., 2009), the RNA-binding protein PHI-62 (RNASEK) (McCormick et al., 2012) and the C-type lectin domain containing protein IRG-7 (Yunger et al., 2017) can modulate DAF-16 function, but their mechanism of action is not fully understood. Altogether, these modifications modulate DAF-16-mediated stress resistance and longevity.

### 1.2. HLH-30 (TFEB)

A key longevity mechanism is the autophagy process (see cellular mechanisms of longevity below) and it is in part modulated by transcription (reviewed in (Lapierre et al., 2015)). A major regulator of autophagy and lysosomal gene expression is the Transcription Factor EB (TFEB), an autophagy enhancer found in *C. elegans* as HLH-30. HLH-30/TFEB is required for the autophagic response to starvation (O’Rourke and Ruvkun, 2013; Settembre et al., 2013), for innate immunity (Visvikis et al., 2014) and for lifespan extension in different long-lived nematode mutants (Lapierre et al., 2013). The nuclear localization of HLH-30/TFEB is modulated via phosphorylation by mTOR (Lapierre et al., 2013; Roczniak-Ferguson et al., 2012; Settembre et al., 2011). In the nucleus, HLH-30/TFEB function is regulated via competition with MXL-3/MAX (O’Rourke and Ruvkun, 2013) and by interaction with proteins of the Mondo-complex (Nakamura et al., 2016). Interestingly, HLH-30/TFEB and DAF-16/FOXO are both required for longevity associated with reduced lipid secretion (Seah et al., 2016), suggesting potential nuclear interactions between these transcription factors. The nuclear export of HLH-30/TFEB is regulated by nuclear export protein XPO-1/XPO1 and selective inhibitors of nuclear export enhance HLH-30/TFEB activity (Silvestrini et al., 2018). Consequently, enhancing lysosomal function pharmacologically via HLH-30/TFEB activation leads to lifespan extension in *C. elegans* (Silvestrini et al., 2018; Wang et al., 2017). Therefore, modulation of HLH-30/TFEB nuclear localization may be an exploitable strategy to stimulate the autophagy/lysosomal pathway and improve somatic maintenance.

### 1.3. PHA-4 (FOXA)

Another member of the forkhead box family of transcription factors, PHA-4(FOXA), was originally identified as a central factor in foregut development (reviewed in (Mango, 2009)) and later found to also be a key transcription factor in lifespan extension upon dietary restriction (Panowski et al., 2007). PHA-4 was also found to be important in the long lifespan of germline-less animals (Lapierre et al., 2011; O’Rourke et al., 2013). The expression of the transcription factors *pha-4* and *skn-1* (see below) can be modulated by micro RNAs miR-71 and miR-228 (Smith-Vikos et al., 2014). In line with its negative role on lifespan, TOR signaling impairs the function of PHA-4 (Lapierre et al., 2011; Sheaffer et al., 2008). During development, PHA-4 binds promoters of multiple genes (Zhong et al., 2010) and affects chromatin dynamics and RNA polymerase function (Fakhouri et al., 2010; Hsu et al., 2015). PHA-4′s role also includes the modulation of the expression of superoxide dismutases and autophagy genes associated with lifespan extension (Lapierre et al., 2011; Panowski et al., 2007).

### 1.4. HIF-1 (HIF1)

The hypoxia-inducible factor-1 (HIF-1) has been linked to lifespan extension in various longevity models (reviewed in (Hwang and Lee, 2011)). For instance, in *C. elegans*, reduction of the conserved acyl-CoA binding protein MMA-1/ACBP-1 (Shamalnasab et al., 2017) or the inhibition of the E3 ligase elongin (Hwang et al., 2015) require HIF-1 activation for lifespan extension. Moreover, in the mitochondrial mutant *isp-1,* where ketoacids levels are elevated (Butler et al., 2013), HIF-1 activity is increased and contributes to the lifespan extension (Mishur et al., 2016). Interestingly, supplementing animals with ketoacid α-ketoglutarate is sufficient to extend lifespan in *C. elegans* by reducing the ability of mitochondria to produce ATP, thereby activating autophagy (Chin et al., 2014). Iron starvation by frataxin suppression also stimulates mitochondrial autophagy (mitophagy) in part via HIF-1 activation (Schiavi et al., 2015). Recent work has uncovered that neuronal HIF-1 modulates serotonin signaling to the intestine, where a xenobiotic response is elicited via HLH-30-regulated expression of flavin-containing monooxygenase 2 (Leiser et al., 2015). Altogether, the requirement of HIF-1 on lifespan extension appears to be context-dependent (Table 1).

### 1.5. HSF-1 (HSF1)

The heat shock transcription factor HSF-1 (HSF1) increases the expression of chaperones in response to various proteotoxic stressors, including but not limited to heat (reviewed in (Li et al., 2017)). More recently, maintenance of cytoskeletal integrity was identified as another mechanism through which HSF-1 increases thermotolerance (Akerfelt et al., 2010; Baird et al., 2014). Under non-stress conditions, HSF-1 regulates developmental and metabolic genes as well as genes involved in collagen biogenesis (Akerfelt et al., 2010; Brunquell et al., 2016). As in other organisms, activation of *C. elegans* HSF-1 upon heat shock involves oligomerization and apparently, changes in posttranslational modifications, including phosphorylation (Anckar and Sistonen, 2011; Chiang et al., 2012a). Reduction of insulin/IGF-1 signaling, but not heat, activates HSF-1 by promoting phosphorylation of DDL-1 (CCDC53) by an unidentified kinase, which leads to destabilization of the DDL-1/DDL-2 (WASH2)/HSB-1 (HSBP1)-complex that inhibits HSF-1. Upon heat shock, at least in larvae, but apparently not in adult worms (Berber et al., 2016), the protein kinase HPK-1 indirectly activates HSF-1 by interfering with inhibitory HSF-1 sumoylation (Das et al., 2017). HSF-1 is also subjected to complex regulation during thermal stress and DR by the integrin-linked kinase PAT-4 (ILK) and the deacetylase SIR-2.1 (SIRT1) (Kumsta et al., 2014; Raynes et al., 2012). While persistent heat stress is unequivocally detrimental to nematode survival, it is interesting to note that intermittent heat shock can extend lifespan via HSF-1 activation (Kumsta et al., 2017).

### 1.6. SKN-1 (NRF2)

Beyond its function in inducing phase II detoxification genes upon oxidative stress, SKN-1 (NRF-2) has been implicated in the response to other stressors such as ER stress and starvation, and in various homeostatic processes even in the absence of stress, such as proteostasis and lipid metabolism (reviewed in (Blackwell et al., 2015)). Interestingly, in the context of reduced IIS, *skn-1* is only required for longevity under conditions that do not induce dauer-like traits (Ewald et al., 2015; Tullet et al., 2008). Under basal conditions, SKN-1 is inhibited by phosphorylation by AKT-1/2, SGK-1 (Tullet et al., 2008) and GSK-3 (An et al., 2005) while upon oxidative stress, SKN-1 is activated by PMK-1/p38 MAP-kinase dependent phosphorylation (Inoue et al., 2005). Apparently downstream of PMK-1, GSK-3 and IIS-signaling, the WD40-repeat protein WDR-23 and the CUL-4/DDB-1 E3 ligase complex modulate SKN-1 activity (Choe et al., 2009). Importantly, a similar WDR23-DDB1-CUL4 axis appears to regulate NRF2 in mammalian cells independently to the previously established KEAP1-CUL3 axis (Lo et al., 2017). SKR-1/2 (orthologues of the mammalian SCF-ubiquitin ligase complex member SKP1) also promote SKN-1 target gene expression upon oxidative stress (Wu et al., 2016a) and were reported to be required for longevity of *daf-2* mutant *C. elegans* (Ghazi et al., 2007). Evidence suggested that DAF-16 is a target of SKR-1/2, although SKN-1 was not examined in this context (Ghazi et al., 2007). Interestingly, a recent study suggested that *skn-1* can be transcriptionally regulated by *daf-16* and that *skn-1* mediated stress resistance may not be necessary for longevity (Tullet et al., 2017). However, whether these regulatory connections are limited to artificial settings such *daf-16* overexpression remains unclear.

### 1.7. Nuclear hormone receptors

Signaling via nuclear hormone receptors affects metabolism, xenobiotic responses, stress resistance and longevity (reviewed in (Hoffmann and Partridge, 2015)). For instance, the nuclear hormone receptor DAF-12 and bile acid like steroids called dafachronic acids (DA) (Antebi, 2013) are required for germline-longevity and metabolomics analyses identified specific DAs as endogenous ligands for DAF-12 (Mahanti et al., 2014; Motola et al., 2006). DA biosynthesis appears to be distributed across several tissues and may include contributions from the somatic gonad, consistent with the notion that DAs contribute to the longevity-promoting signal from this tissue (Antebi, 2013). While exogenous DA can increase the lifespan of somatic gonad-deficient, but not of somatic gonad-competent, germline-less animals (Yamawaki et al., 2010), DA’s ability to extend wildtype lifespan is controversial (Gerisch et al., 2007; Yamawaki et al., 2010), and this requirement may be more robust at 25 °C (Li et al., 2015). Moreover, there are conflicting reports on elevated DA-levels in germline-deficient *glp-1* animals (Li et al., 2015; Shen et al., 2012). Recently, DA has also been implicated in DR-induced longevity, but in this context, DA signals through the nuclear hormone receptor NHR-8, rather than DAF-12, to repress *let-363 (mTOR)* mRNA-levels (Antebi, 2013; Thondamal et al., 2014). Lifespan extension by DR was also linked to NHR-62 (HNF4a)-mediated gene regulation (Heestand et al., 2013).

NHR-80 is another nuclear hormone receptor whose elevated expression is required for the longevity of germline-less animals (Goudeau et al., 2011). This NHR-80 upregulation is only partially dependent on *daf-12* and *daf-16*, (Goudeau et al., 2011). Moreover, NHR-80 has been reported to physically interact with NHR-49 (HNF4/PPARa) (Pathare et al., 2012) and NHR-49 is also upregulated in germline-less *glp-1* animals, however, dependent on *daf-16* (Ratnappan et al., 2014). In addition to its role in modulating expression of β-oxidation genes (Van Gilst et al., 2005a,b), NHR-49 has recently been shown to mediate a transcriptional response to fasting and oxidative stress (Goh et al., 2018). Endogenous ligands for NHR-80 and NHR-49 are currently unknown, although the monounsaturated fatty acid oleic acid (OA) is a candidate NHR-80 ligand (Goudeau et al., 2011).

## 2. Longevity-regulating signals

Small molecules and endocrine signals have been linked to longevity and mediate changes in different signaling pathways with effects on downstream transcription factors and effector mechanisms. Recent studies in longevity regulation have focused on cell-autonomous and cell-non-autonomous signals to modulate organismal lifespan.

### 2.1. Neuroendocrine signals

Observations such as lifespan modulatory effects of sensory perception through olfactory and gustatory neurons (Alcedo and Kenyon, 2004; Apfeld and Kenyon, 1999) or inhibition of the DR-induced longevity response of peripheral tissues by diffusible compounds from the bacterial food source indicated a role for (neuro)endocrine signals in lifespan regulation (Bishop and Guarente, 2007; Smith et al., 2008). A recent study suggested that upon DR, DAF-7 (TGFβ) secreted by ASI neurons constitutes a pro-longevity signal that contributes to intestinal DAF-16 activation (Fletcher and Kim, 2017). Moreover, an age-associated decrease in DAF-7 levels may explain why *C. elegans*’ sensitivity to the longevity-promoting effects of DR decreases over time (Fletcher and Kim, 2017). In contrast, in fed animals, lifespan is extended when DAF-7 signaling is suppressed by branched chain amino acids (BCAAs) from the periphery that activate *let-363* in ASI neurons (Mansfeld et al., 2015). Thus, although global inhibition of TOR extends lifespan, activating TOR can also exert this effect, when occurring in specific neurons (Mansfeld et al., 2015). Supplementation with the BCAT-1 (branched-chanin-amino-acid aminotransferase) substrate L-leucine or RNAi knockdown of *bcat-1* or *hlh-15* (NHLH1), which regulates *bcat-1* transcription, is sufficient to extend *C. elegans* lifespan dependent on *daf-16* and *hsf-1* (Mansfeld et al., 2015). An independent study also reported that *daf-7*’s role in lifespan regulation is dependent on feeding state and suggested that combinatorial expression of *daf-7* and the serotonin biosynthetic enzyme *tph-1* (tryptophan hydroxylase) encodes food availability *in vivo* (Entchev et al., 2015). On the other hand, the ASI and ASJ-derived insulin like peptide INS-6 apparently mediates a bacterial food-derived anti-longevity signal that is sufficient to block DAF-16 nuclear accumulation in peripheral tissues and, partially, longevity in otherwise food-restricted *C. elegans* (Artan et al., 2016).

### 2.2. Reactive oxygen species

ROS have been implicated in aging because of their potential to cause macromolecular damage, (Finkel, 2011). Yet, treatment with low doses of ROS-generators such as paraquat and jugulone can lead to lifespan extension dependent on *hif-1* and *aak-2* (AMPKα) or on *daf-16* and *sir-2.1*, respectively (Heidler et al., 2010; Hwang et al., 2014; Yang and Hekimi, 2010). Within the cell, ROS are generated as a by-product during mitochondrial electron transport and certain enzymatic reactions, but also as a primary product from professional ROS generating enzymes such as NADPH-oxidases (Finkel, 2011). Apart from dose, the localization of ROS generation within the cell and the precise ROS species may be important factors that determine the cellular and organismal outcome of ROS presence (Heidler et al., 2010; Lee et al., 2010; Yang and Hekimi, 2010). Of note, superoxide anions, a ROS species that cannot cross biological membranes (Krause, 2007), appears to be particularly important in at least some *C. elegans* longevity paradigms, such as *daf-2*, the mitochondrial mutants *nuo-6* and *isp-1* (Yang and Hekimi, 2010) and germline-less worms (Wei and Kenyon, 2016). Thus, the localization of professional superoxide generators such as NADPH-oxidases and, as proposed recently, globins, and eventually, their interplay with superoxide dismutases, allow to spatially control redox signaling (De Henau et al., 2015; Krause, 2007; Schaar et al., 2015). ROS originating from mitochondria or from the ER through the NADPH-oxidase BLI-3 (DUOX1/2) cause inhibitory sulfenylation of the ER-stress sensing kinase inositol requiring enzyme-1 (IRE-1) (Hourihan et al., 2016), consequently inhibiting the UPR^ER^ (see below) and inducing a p38/SKN-1 mediated antioxidant response. *bli-3*, ROS and *skn-1* also mediate lifespan extension in response to loss of *memo-1* (ortholog of mammalian mediator of ErbB2 driven cell motility) (Ewald et al., 2017) and enhanced pathogen resistance upon elevated proline catabolism (Liang et al., 2013; Tang and Pang, 2016). Moreover, a transient ROS-signal generated by enhanced proline catabolism in *daf-2* worms contributes to their longevity (Zarse et al., 2012). Therefore, the impact of ROS production on redox balance and signaling in different compartments of the cell remains to be elucidated. In summary, depending on the context, ROS are not only damaging agents that promote aging, but are also emerging as important signaling molecules that can promote longevity.

### 2.3. Hydrogen sulfide (H_2_S)

Increased endogenous H_2_S production has been reported to be critical for various DR-induced benefits in diverse organisms, including longevity in *eat-2* mutant *C. elegans* (Hine et al., 2015). Moreover, H_2_S has been implicated in the longevity of *glp-1* worms (Wei and Kenyon, 2016) and exogenous H_2_S extends worm lifespan (Miller and Roth, 2007). H_2_S is generated during sulfur amino acid metabolism and acts as a gaseous messenger molecule that modulates cellular signaling through protein sulfhydration and other mechanisms (Kabil et al., 2014; Paul and Snyder, 2012). The ability of worms to tolerate low levels of H_2_S depends on *skn-1* and *hif-1* and indeed, in germline-deficient worms, H_2_S, rather than ROS, appears to activate *skn-1* (Budde and Roth, 2010; Miller and Roth, 2007; Topalidou and Miller, 2017; Wei and Kenyon, 2016). Interestingly, *hif-1* is not required for the H_2_S-mediated longevity of *eat-2* and *glp-1* worms (Table 1). Recently, the sulfide-quinone oxidoreductase SQRD-1, which mediates H_2_S benefits in cultured cells (Hine et al., 2015) has also been implicated in maintaining proteostasis in H_2_S-exposed worms (Horsman and Miller, 2016). Whether increased levels of H_2_S are a broad mechanism for longevity remains to be determined.

### 2.4. Nutrient and energy sensors

The best-established links between metabolism and aging stem from the discovery that the major amino acid sensor and growth regulator, the mechanistic Target Of Rapamycin (mTOR) as well as the energy sensor AMP-activated protein kinase (AMPK) modulate lifespan across phyla (Burkewitz et al., 2014; Hansen and Kapahi, 2010; Lapierre and Hansen, 2012; Laplante and Sabatini, 2012). Lifespan extension upon deficiency in the ribosomal protein S6 kinase, a key TOR-complex 1 substrate (Kapahi et al., 2010) was recently reported to require the arginine kinase ARGK-1 (ortholog of creatine kinase) (McQuary et al., 2016). *argk-1* is dispensable for *daf-2* and *eat-2* longevity and appears to function together with *aak-2*/AMPK (McQuary et al., 2016). Yet, the precise regulatory mechanisms that link ARGK-1 activation to RSKS-1 (S6K) inactivation and AAK-2 activation remain to be determined. AAK- and its substrate, the CREB-regulated transcriptional co-activator CRTC-1 (Mair et al., 2011) were also implicated in longevity of ETC-compromised by activating the homeobox transcription factor CHE-23 (EMX1/2) and CEP-1/p53 (Chang et al., 2017a). How AAK-2 modulates CEP-1 activity has not been elucidated but it is interesting to note that mammalian p53 may be a substrate of AMPK (Jones et al., 2005). Altogether, energy levels and nutrient status are key molecular cues for cells to initiate stress resistance and survival mechanisms that affect lifespan.

## 3. Cellular processes mediating longevity

Longevity-associated transcription factors modulate genes that drive the activity and efficiency of complex processes in the cell, which translates into improved somatic maintenance. Major proteostatic pathways have been linked to lifespan extension and include protein degradation pathways such as the autophagy/lysosomal pathway and the ubiquitin proteasome system as well as protein metabolism in the endoplasmic reticulum and the mitochondria. Aging animals are characterized by proteostatic decline (Ben-Zvi et al., 2009), altered protein turnover (Dhondt et al., 2017) and the accumulation of insoluble proteins (Reis-Rodrigues et al., 2012). A cell’s response to the global loss of protein stability and solubility during aging includes enhanced autophagic degradation (Chang et al., 2017b; Chapin et al., 2015; David et al., 2010), disaggregation (Nillegoda et al., 2015), but also, intriguingly, packaging of aggregating proteins into chaperone-enriched aggregates (Moll et al., 2016; Walther et al., 2015). Here, we describe new findings in cellular processes with benefits on proteostasis, stress resistance and lifespan.

### 3.1. Autophagy

mTOR and AMPK modulate the process of autophagy, a recycling mechanism that results in the sequestration and lysosome-mediated breakdown of damaged macromolecules and organelles into basic components that become substrates for various biogenic pathways. This cellular “rejuvenation” pathway has emerged as a central mechanism in the ability of cells to maintain proteostasis, signaling and transcriptional signatures associated with survival. The ability of cells to engage and maintain autophagic flux is in part governed by transcription factors such as HLH-30/TFEB, PHA-4/FOXA and DAF-16/FOXO that translocate to the nucleus to enhance autophagy and lysosomal gene expression (Lapierre et al., 2015). More recently, selective autophagy of particular cellular cargo has been linked to longevity. Breakdown of compromised mitochondria by mitophagy has been shown to be important for prolonged lifespan in the worm (Palikaras et al., 2015). Autophagy stimulation can be recapitulated using pharmacological agents against upstream negative regulators (Galluzzi et al., 2017a, b). For instance, inhibitors of mTOR can activate autophagy and lysosomal biogenesis in part via HLH-30/TFEB activation (Roczniak-Ferguson et al., 2012; Settembre et al., 2011). Specifically, targeting the activity of TFEB has emerged as a viable option to stimulate autophagy. However, pharmacological targeting of TFEB has been particularly challenging since several drugs improving the nuclear localization of TFEB and lysosomal biogenesis have lysosomotropic properties that inhibit mTOR and impair lysosome function in cells (Lu et al., 2017). Nonetheless, new small molecule activators of autophagy via TFEB activation are emerging (Wang et al., 2017; Silvestrini et al., 2018). Other transcription factors, such as HSF-1, have been shown to modulate autophagy gene expression in the context of heat shock (Kumsta et al., 2017), suggesting that autophagy induction is a converging process cells use to maintain the soma under various extrinsic stresses. Lysosome biogenesis via expression of lysosomal proteins and degradation enzymes is increased in long-lived animals (Florez-McClure et al., 2007; Lapierre et al., 2013; Li et al., 2016; McColl et al., 2008, 2010). Lysosomal pH in the intestine can be modulated by DAF-16/FOXO-mediated transcriptional upregulation of proton v-ATPase genes (Baxi et al., 2017). A recent study highlighted that induction of the lysosomal proton V-ATPase subunit VHA-13 during fertilization is sufficient to efficiently clear damaged proteins in oocytes (Bohnert and Kenyon, 2017), demonstrating that lysosomal enhancement can restore proteostasis. Proper autophagosome assembly is crucial in the response to stress and in longevity. Longevity of *eat-2, glp-1, rsks-1* and *daf-2* mutant worms is dependent on the autophagy machinery (Lapierre et al., 2015). Specifically, autophagy in chemosensory neurons mediates signaling to the intestine (Minnerly et al., 2017) and autophagy in intestinal cells is essential for the integrity of the worm gut (Gelino et al., 2016). These data in the worm link the new molecular understanding of the autophagy machinery with animal physiology and longevity.

The relationship between lipid metabolism, autophagy and lifespan is emerging as a key interaction in longevity (Hansen et al., 2013; Lapierre et al., 2012). Autophagy is required for the accumulation of neutral lipids in the intestine of nematodes (Lapierre et al., 2013). Lipid composition in membranes correlates with longevity (Hulbert et al., 2007) and biogenesis of particular lipids correlates with long lifespan in *C. elegans* (Shmookler Reis et al., 2011). Aging markedly changes overall lipid composition and leads to accumulation of very long chain fatty acids (Gao et al., 2017). Recent evidence points to a potential role for oleic acid in longevity (Han et al., 2017), although supplementation experiments have not robustly shown lifespan extension (Goudeau et al., 2011). Regulated lipid turnover has been linked to long-term survival (Narbonne and Roy, 2009). In particular, enhanced lysosomal lipolysis has been shown to extend lifespan (Lapierre et al., 2011; Wang et al., 2008) and to mediate lipid signals driving nuclear hormone receptor (NHR) signaling (Folick et al., 2015; Seah et al., 2016). Indeed, NHR signaling is a central longevity mechanism in different long-lived models (Goudeau et al., 2011; Heestand et al., 2013; Ratnappan et al., 2014) (Table 1). Fatty acids such as oleylethanolamine, derived from lysosomal lipolysis and transported by lipid binding proteins such as LBP-8, have been linked to NHR signaling longevity (Folick et al., 2015). However, lipid signals have not been systematically addressed in the context of aging. Larger polyunsaturated lipids, such as omega-3 and −6 fatty acids have been linked to NHR signaling, autophagy activation and germline signaling (Lynn et al., 2015; O’Rourke et al., 2013; Qi et al., 2017). In addition, cholesterol can drive DAF-16/FOXO activity via lipid-binding protein NSBP-1 (Cheong et al., 2013; Ihara et al., 2017). These studies warrant further understanding of the integration of various fatty acids and sterols with signaling and proteostatic pathways during the process of aging.

Long-lived animals coordinate their lipid stores with lysosomal lipolysis by reducing the expression of large lipid transporters called vitellogenins (DePina et al., 2011; Dong et al., 2007; Murphy et al., 2003; Seah et al., 2016). In turn, lipids bound for yolk protein biogenesis are re-routed to storage, remodeling, and signaling associated with autophagy and somatic maintenance (Seah et al., 2016). Lipid redistribution is accompanied by improvements in lysosome function and nuclear hormone receptor signaling. While enhanced vitellogenesis is not detrimental in *C. elegans* (Seah et al., 2016), rearrangement of lipid stores by reduced vitellogenesis is essential for the ability of animals to survive starvation (Harvald et al., 2017). Some, but not all long-lived animals have enhanced lipogenesis that leads to increased lipid storage (Amrit et al., 2016; Perez and Van Gilst, 2008). Animals unable to concomitantly increase lipogenesis or redistribute lipids have decreased lipid stores when autophagy and lysosomal lipolysis are enhanced (Schiavi et al., 2013; Wang et al., 2011). Interestingly, lipid droplet biogenesis has recently been linked to longevity via modulation of the intake of fatty acid to mitochondria (Nguyen et al., 2017). These findings point to an intra-organelle integration involving lipid droplet biogenesis and mitochondrial function that can be modulated by the autophagy/lysosomal pathways and nuclear hormone signaling.

### 3.2. Unfolded protein response of the endoplasmic reticulum

The endoplasmic reticulum manages biochemical changes in its lumen via the unfolded protein response (UPR^ER^). This multibranch pathway has a number of ER luminal sensors that transmit the information resulting in gene expression changes that reset ER homeostasis. The sensor proteins are IRE-1, PERK, and ATF-6. The ER transmembrane stress sensor IRE-1 (Chen and Brandizzi, 2013) modulates the UPR-related transcription factor XBP-1 through splicing of its mRNA to permit synthesis of the functional transcription factor. Together with its role in the antioxidant defense (Hourihan et al., 2016), as discussed above, these combined functions place IRE-1 into the center of cellular homeostasis and stress response. It is particularly interesting that IRE-1 can receive distinct inputs that result in different downstream consequences. Of note, IRE-1 signaling to SKN-1 or via the UPR both encode a stress signal and the respective responses have been linked to longevity. Interestingly, a recent study likewise linked the stress response via *skn-1* and *ire-1* with enhanced fitness (Mark et al., 2016). Vitamin D promotes protein homeostasis and longevity by triggering *skn-1* and *ire-1* UPR branch pathways. These data further support the concept of ER hormesis and show that a certain tone in UPR^ER^ signaling can be a mechanism for enhanced fitness and longevity. Hormesis is an adaptive response to a low level of detrimental stress that triggers an adaptation which subsequently leads to stress resistance and robustness. Conceptually, this is akin to mitohormesis, the process by which low doses of ROS have beneficial effects on mitochondrial function (Schulz et al., 2007). ER stress signaling can thus be a trigger for an adaptive response that mediates longevity in the worm. Upon stress, PERK phosphorylates eIF2α, which reduces initiation of mRNA translation and leads to expression of ATF4 that participates in nuclear gene expression changes enhancing ER protein folding capacity. ATF-6 is likewise an ER luminal sensor that becomes processed in the Golgi apparatus upon stress to directly activate expression of gene that mitigate ER stress.

While the role of the UPR^ER^ in stress adaptation is intriguing, it remains elusive if ER signaling pathways might also be involved in reversing aging. A recent study showed that larval starvation in the worm results in a number of age-associated phenotypes, which are reversed upon return of the animals to food (Roux et al., 2016). Excitingly, this “correction” of age-associated phenotypes, with the exception of protein aggregates, was dependent on IRE-1. This points to two possible roles of IRE-1 in longevity. For instance, a signal of ER stress and UPR^ER^ might be required for normal homeostasis. Alternatively, during development IRE-1 might have functions that are entirely distinct from ER sensing and downstream signaling. Certainly, future work will address the question of whether age-associated phenotypes will also be reversible in the adult worm, and whether IRE-1 might be involved in this process.

The FOXO transcription factor DAF-16 also promotes ER homeostasis. Specifically, DAF-16 releases ER stress by enhancing autophagy-mediated degradation independently of IRE-1 UPR-pathway activated genes, such as ERAD genes (Safra et al., 2014). While ER stress does not directly trigger DAF-16, its activity promotes ER homeostasis. In addition, DAF-16 interacts with the UPR^ER^-activated transcription factor XBP-1 (Henis-Korenblit et al., 2010). This orchestrated function of DAF-16 clearly demonstrates the critical role of the ER in longevity. Several additional observations support a link between UPR^ER^ signaling and longevity. Mutant toxic proteins themselves initiate an UPR (Fardghassemi et al., 2017; Singh and Aballay, 2017). However, is a reduction of protein misfolding sufficient to extend lifespan? Forward genetic approaches were used to directly identify factors that simultaneously enhance stress resistance and extend lifespan. Heat, which leads to protein folding stress, can be a proxy for protein aggregation stress. A screen for resistance to heat stress identified novel alleles in many longevity genes, including the *daf-2* gene (Munoz and Riddle, 2003). Importantly, protein aggregates accumulate with age in *C. elegans* (David et al., 2010), and human disease-associated toxic proteins aggregate in aging transgenic worms (Morley et al., 2002). A screen for resistance to tunicamycin, which triggers ER stress through inhibition of N-glycosylation, yielded a large number of resistant and long-lived mutant strains (Denzel et al., 2014). Of note, activation of the metabolic hexosamine pathway, which provides substrates for N-glycosylation, extended lifespan through engagement of autophagy, ERAD, and mild upregulation of proteasome activity. This suggested that degradation of proteins can suffice to extend lifespan in the absence of disease linked aggregation prone proteins. Moreover, it was found that compounds that directly bind to amyloid protein aggregates can extend worm lifespan (Alavez et al., 2011). This effect was *hsf-1* and *skn-1* dependent and thus it is unclear if it results from direct action on protein aggregates, or from altered stress signaling.

In addition to supporting the formation of autophagosomes, the ER is the site of *de novo* lipid droplet biogenesis, which is an essential process in the worm (Choudhary et al., 2015). Consistently, the ER is also a site of lipid and membrane composition sensing. Lipid dis-equilibrium is *per se* sufficient to trigger the UPR in the absence of disrupted protein folding (Hou et al., 2014). Moreover, IRE-1 acts as a direct sensor for ER membrane composition (Promlek et al., 2011; Volmer et al., 2013). Thus, IRE-1 is positioned at a very interesting cross road of protein and lipid homeostasis. How downstream signaling integrates and differentiates between the two processes will be exciting field of future research.

The UPR^ER^ was traditionally considered a cell-autonomous mechanism maintaining cellular protein homeostasis. Recent data, however, have expanded this view by demonstrating cell-non-autonomous regulation of the UPR^ER^. Ectopic expression of spliced *xbp-1* in the worm’s nervous system triggered peripheral expression of the UPR target gene *hsp-4* and extended lifespan (Taylor and Dillin, 2013). Interestingly, this effect was *ire-1* dependent, demonstrating that the peripheral response requires the entire arm of the UPR^ER^, including the stress sensor. This work suggests the presence of yet unidentified neuroendocrine signaling molecules that mediate the cell-nonautonomous effect on proteostasis (Taylor et al., 2014).

### 3.3. The ubiquitin-proteasome system (UPS)

Cellular protein turnover is mediated in part by the ubiquitin-proteasome system, in which polyubiquitylation factors identify and mark aberrant proteins for degradation. Does enhancing proteasome function prevent organismal aging? Evidence of lifespan extension through induction of proteasome subunit expression, assembly or activity suggest that this is indeed the case (Chondrogianni et al., 2015; Vilchez et al., 2012). In addition, treatment with the proteasome activating compound 18α-Glycyrrhetinic Acid was shown to extend lifespan in the worm (Papaevgeniou et al., 2016). In line with this, loss of proteasome activity explains the lifespan reduction in glucose-fed animals (Fitzenberger et al., 2013). In addition, protein aggregates related to neurodegeneration were shown to block proteasome activity (Ayyadevara et al., 2015) and proteasome inhibition elicited a stress response via SKN-1 and autophagy (Keith et al., 2016; Lehrbach and Ruvkun, 2016). The UPS pathway was linked to the longevity-related insulin signaling longevity pathway. Surprisingly, the ubiquitin ligase CHIP regulates the insulin receptor DAF-2 directly by monoubiquitination and subsequent endocytic-lysosomal degradation. CHIP activity thus maintains low DAF-2 cell surface abundance, low insulin signaling tone, consequently affecting longevity (Tawo et al., 2017). With increased demand on the UPS with aging, CHIP activity towards DAF-2 is reduced, resulting in enhanced DAF-2 expression with age. This work suggests a cross talk between protein aggregates and DAF-2 expression that results in a self-accelerating cycle between protein aggregates that eliminates the protective low insulin signaling tone. Unexpectedly, recent studies of long-lived *daf-2* animals have demonstrated that enhancement of proteasomal function is not necessarily a common mechanism for longevity. Lower proteasome activity was observed in *daf-2* animals as well as reduced protein turnover (Stout et al., 2013). In addition, the half-life of proteins in *daf-2* animals is extended (Depuydt et al., 2016; Dhondt et al., 2016; Visscher et al., 2016), which suggests that long lifespan may be achieved by globally enhancing protein stability thereby reducing the global requirement for rapid turnover and synthesis.

### 3.4. The mitochondrial unfolded protein response (UPR^mt^)

Perturbations in mitochondrial protein homeostasis triggers the mitochondrial unfolded protein response that induces nuclear gene expression changes to cope with the stress. This results in expression of mitochondria-associated protective genes to restore mitochondrial function (Qureshi et al., 2017). Although first described in mammalian cells, key components of the UPR^mt^-pathway have been identified in *C. elegans* (Qureshi et al., 2017), including the mitochondrial quality control protease CLPP-1 (CLPP), the peptide transporter HAF-1 (ABCB10), the transcription factors ATFS-1 (ATF4/5) and DVE-1 (SATB1/2), and the ubiquitin-like protein UBL-5 (UBL5), (Pellegrino et al., 2013; Qureshi et al., 2017). Complementary to ATFS-1 mediated changes in transcription, the eIF2α kinase GCN-2 lowers cytosolic protein translation when activated by increased ROS from dysfunctional mitochondria (Qureshi et al., 2017). Initial studies implicating the UPR^mt^ (Durieux et al., 2011), or more broadly, mitonuclear imbalance (Houtkooper et al., 2013), into longevity of worms with compromised mitochondrial function were subsequently challenged (Bennett and Kaeberlein, 2014; Bennett et al., 2014). Indeed, several conditions have been identified in which mitochondrial perturbation shortens lifespan in the presence of an active UPR^mt^ (Bennett and Kaeberlein, 2014). In some cases, induction of the UPR^mt^ apparently even confers a disadvantage, for example in a short-lived heteroplasmic strain (Liau et al., 2007), where a constitutively active UPR^mt^ contributes to maintenance and propagation of mutated mitochondrial genomes (Gitschlag et al., 2016; Lin et al., 2016).

Recent work identified additional regulators of the UPR^mt^ and of longevity-associated factors upon mitochondrial impairment in *C. elegans*. Mitochondrial stress induces chromatin changes dependent on the apparently nematode-specific protein LIN-65 and the H3K9me2-forming methyltransferase MET-2 (SETDB1) (Tian et al., 2016) (Table 2). Moreover, the H3K27me2/3 demethylases JMJD-1.2 (PHF8) and JMJD-3.1 (JMJD3) strongly contribute to longevity of ETC-compromised, but not of *eat-2* animals (Merkwirth et al., 2016). Interestingly, only *jmjd-3.1* was required for *glp-1* (Labbadia and Morimoto, 2015) and (partially) *daf-2* longevity (Merkwirth et al., 2016). Of note, positive correlations between PHF8/JMJD3 and UPR^mt^ signaling mediators/targets are also observed in murine tissues (Merkwirth et al., 2016). On the other hand, the transaldolase TALD-1 and other pentose phosphate pathway enzymes, whose knockdown extends *C. elegans* lifespan, were identified as suppressors of the UPR^mt^ (Bennett et al., 2017). Another recent study (Munkacsy et al., 2016) described a novel pathway that is activated upon disruption of mitochondrial function that contributes to the extended lifespan of ETC defective animals and comprises the kinases DLK-1 (MAP3K12), SEK-3 (MAP2K4) and PMK-3 (MAPK14) and the reporter gene *Ptbb-6*::*GFP*. ETC-knockdown in the nervous system increases lifespan and induces the UPR^mt^ in a distant issue, the intestine, suggesting an endocrine signal (“mitokine”) to coordinate mitochondrial stress signaling and eventually lifespan across tissues (Durieux et al., 2011). A recent study from the same group expanded this cell non-autonomous activation of the UPR^mt^ to neuronal stress upon polyQ-expression (Berendzen et al., 2016). Among other factors, UPR^mt^ induction in this context was dependent on the neuro-transmitter serotonin. Serotonin was also required to transmit a peripheral UPR^mt^ activating signal upon other forms of neuronal stress (Berendzen et al., 2016), but whether it also transmits the lifespan-modulatory signal when the neuronal ETC is impaired has not been explicitly tested. Of note, serotonin also mediates a cell-nonautonomous signal from neurons to the intestine which stabilizes HIF-1 (Leiser et al., 2015).

### 3.5. Heat-shock response

A major player in the proteostasis machinery is the heat shock response. Orchestrated by the key regulator HSF-1, the heat shock response is critical for maintaining homeostasis during aging. Impressive studies have shown how the heat shock response declines precipitously at early adult stages in the worm (Ben-Zvi et al., 2009; Labbadia and Morimoto, 2015), positioning a decline in proteostasis as a very early event in aging in the worm. Recently, they were able to identify suppressors of this phenotype through forward genetic screens and demonstrated that a reduction in mitochondrial ETC activity maintains the heat shock response (Labbadia et al., 2017). This work sheds light on an interesting interplay between mitochondrial activity and cytosolic protein homeostasis. While reduced ETC function has long been associated with longevity, it had not been known that this involves a downstream function of HSF-1, thus linking two major longevity pathways.

In further support of this concept, depletion of a major UPR^mt^ transcriptional target, the mitochondrial chaperone *hsp-6*, triggers a stress response in the cytosol (MCSR: mitochondrial to cytosolic stress response) dependent on multiple UPR^mt^-mediators and on the key transcriptional regulator of the cytosolic heat shock response, *hsf-1* (Kim et al., 2016). Moreover, *hsp-6* depletion triggered the *dve-1* and *hsf-1* dependent expression of lipid metabolic genes, which are not induced under conditions that activate only *dve-1* or *hsf-1*. MSCR induction improved cytosolic protein homeostasis not just in *C. elegans* but also in a human cell culture model (Kim et al., 2016). Of note, although *hsp-6* depletion/MSCR induction apparently has beneficial effect on proteostasis in polyQ-challenged animals, lifespan of wildtype worms is shortened by *hsp-6* RNAi (Kimura et al., 2007)

### 3.6. Protein synthesis

Reduced protein synthesis is a consequence of a number of longevity interventions, including genetic models of longevity in the worm such as the *eat-2* DR model, or the inhibition of TOR (Hansen et al., 2007). However, reduced protein synthesis appears to be *per se* sufficient for lifespan extension. A first indication of this came from initial RNAi longevity screens (Hamilton et al., 2005; Hansen et al., 2007; Lee et al., 2003) that found that knockdown of a number of ribosomal and translation genes resulted in lifespan extension. Reducing translation improves all-over robustness, for example under conditions of ER stress (Howard et al., 2016), and is a characteristic of long-lived *daf-2* animals (Depuydt et al., 2013). Further reducing translation in *daf-2* animals leads to extreme longevity (Chen et al., 2013). Protein synthesis reduction via RNA polymerase PolII inhibition can also mediate lifespan extension (Filer et al., 2017). Moreover, genetic and pharmacological inhibition of mRNA translation extends worm lifespan (Cattie et al., 2016; Syntichaki et al., 2007; Takauji et al., 2016). Interestingly, proteome stability is also sensitive to nascent peptide-ribosome interactions (Kirstein-Miles et al., 2013) as well as to ribosomal dynamics governed by codon translation optimization (Nedialkova and Leidel, 2015).

Why does reduced protein synthesis extend lifespan? One explanation is the reduced demand on the protein folding machinery. Age-dependent changes in protein abundance contribute to protein aggregation as abundant proteins strongly contribute to protein aggregates (Walther et al., 2015). Globally reducing protein synthesis might thus prevent such catastrophic shift in solubility. Reducing load on the protein homeostasis system via reducing protein synthesis, might thus delay protein misfolding by improving translation fidelity and chaperone availability (Hansen et al., 2007; Pan et al., 2007; Syntichaki et al., 2007). This is consistent with the disposable soma theory of aging: fast growth in early life is beneficial and protein misfolding is readily suppressed in young animals due to efficient and responsive proteostatic mechanisms (Kirkwood, 2005). Thus, there is no trade-off in young animals. As animals age, however, protein folding capacity shrinks while the proteome composition shifts significantly, and proteins form insoluble aggregates. With reduced protein synthesis, this effect might be delayed.

In addition, there is a signaling response to reduced protein synthesis. During genetic inhibition of mRNA translation, there is a specific response of the SKN-1 transcription factor (Li et al., 2011) that results in the expression of cytoprotective genes, including *atf-5* and *haf-7*. This suggests that reducing protein synthesis is not only *per se* protective but also triggers a signaling response via SKN-1 that boosts robustness. Similarly, while eIF2α phosphorylation inhibits protein synthesis, it also triggers the ATF-5-dependent transcriptional response. ATF-5 is thus the transcriptional output of the PERK arm of the UPR^ER^. The mammalian homologue ATF4 initiates expression of genes involved in oxidative stress and amino acid metabolism, as well as apoptosis, and the yeast homologue GCN4 is involved in caloric restriction and amino acid starvation. Worm ATF-5 target genes have not been specifically addressed.

ER stress triggers the phosphorylation of eIF2α by the ER kinase PERK. eIF2α is the master regulator of the integrated stress response, which, in the worm, also receives input by general control non-derepressible 2 (GCN-2) kinase that signals amino acid shortage and mitochondrial stress. eIF2α is a critical component of cap-dependent mRNA translation machinery and its phosphorylation leads to reduced levels of protein synthesis (Pakos-Zebrucka et al., 2016). In the mammalian system, upstream open reading frame (uORF) regulated transcripts become expressed under these conditions, most importantly the bZIP transcription factor ATF4, which is a homolog of the yeast GCN4 (Vattem and Wek, 2004).

Another aspect of protein synthesis that has emerged recently relates to the roles of splicing factors in the specific and global modulation of proteomes (Heintz et al., 2017; Tabrez et al., 2017) as well as RNA quality control pathways (Son et al., 2017) and nucleoli formation (Tiku et al., 2016). How protein synthesis rates and overall proteostasis are modulated at the RNA level to provide cellular conditions conducive for longevity remains an important area of research and is bound to continue to yield interesting clues on the rate of aging.

## 4. Epigenetic modifications associated with lifespan

Epigenetic changes, i.e. changes in histone post-translational modification patterns, DNA methylation and chromatin remodeling have been proposed as a hallmark of aging (Lopez-Otin et al., 2013). Studies in *C. elegans* identified several chromatin modifiers that influence lifespan, in some cases even in subsequent generations. As many epigenetic regulators are conserved, these insights from *C. elegans* may be broadly applicable.

### 4.1. Histone expression and modifications, and nucleosome positioning

Beyond sirtuins, a family of NAD ^+^-dependent histone deacetylases whose longevity-promoting function in *C. elegans* has been challenged (although evidence for beneficial effects on mammalian lifespan and healthspan is substantial), other modifiers of histone methylation have been implicated in *C. elegans* lifespan regulation (Giblin et al., 2014; Imai and Guarente, 2016) (Table 2). While marked changes in global levels of euchromatin (active) methyl marks have not been observed, heterochromatin (repressed) marks appear to decrease as *C. elegans* ages (Benayoun et al., 2015). Although these and other findings in *C. elegans* are consistent with the notion that loss of heterochromatin and redistribution of euchromatin is detrimental to a long lifespan (Benayoun et al., 2015), the picture is not entirely uniform. For example, decreasing levels of the H3K27me3 demethylase UTX-1 extends worm lifespan (Benayoun et al., 2015; Jin et al., 2011; Maures et al., 2011; Ni et al., 2012), while decreasing levels of the apparent H3K27me3-forming methyltransferase MES-2 was reported to not shorten, but rather, to extend worm lifespan (Benayoun et al., 2015; Ni et al., 2012). The same pattern is observed for depletion of regulators of another repressive mark, H3K9me3, with the caveats that the demethylase JMJD-2 also appears to deplete the activating H3K36 mark and that the function of SET-9/26 as H3K9me3 generating methyl-transferases is not firmly established (Greer et al., 2014; Greer and Shi, 2012; Ni et al., 2012). Integrating different studies is further complicated by different experimental conditions in the respective studies, such as the use of FUDR or of the sterile *glp-1(e2144ts)* strain. Modifiers of the activating H3K4me3 influence *C. elegans* lifespan through lipid metabolism, characterized by increased accumulation of lipids, particularly lipids containing monounsaturated fatty acids (Han et al., 2017). On the other hand, the activating H3K36me3 mark has been suggested to promote longevity by restricting gene expression changes and suppressing cryptic transcription (Pu et al., 2015; Sen et al., 2015). Of note, methyltransferases and demethylases frequently possess a broad substrate specificity and many studies do not formally rule out the possibility that the identified regulators modulate lifespan, at least in part, through targets other than histone proteins (Greer and Shi, 2012).

Some regulators of histone methylation have been reported to interact with well-established longevity pathways. For example, *utx-1* knockdown extends lifespan of *eat-*2 and of wildtype animals in a *daf-16* dependent manner but does not increase *daf-2* longevity (Jin et al., 2011; Maures et al., 2011; Ni et al., 2012). Moreover, the *daf-2* gene appears to be a direct UTX-1 methylation target in both worms and mammalian cells (Jin et al., 2011; Maures et al., 2011). On the other hand, lifespan extension by *set-9/26* or *ash-2* knockdown was at best partially dependent on DAF-16 (Greer et al., 2010; Ni et al., 2012). Furthermore, multiple methyltransferases and demethylases (Table 2) have been examined for their lifespan-regulatory effect in germline-deficient *glp-1* worms. Ability or inability of particular knockdowns to extend *glp-1* lifespan has been taken as evidence that these factors modulate lifespan by acting in the soma/germline (Greer et al., 2010; Hamilton et al., 2005; Jin et al., 2011; Maures et al., 2011; Ni et al., 2012). However, these findings are further consistent with the notion that these knockdowns trigger lifespan-extending mechanisms that are not yet, or already, active in long-lived *glp-1* worms.

Reduced core histone expression during aging has been observed in multiple species, including *C. elegans* (Benayoun et al., 2015) and has been proposed to contribute to aging by precluding proper maintenance of chromatin structure, thus broadly dysregulating transcription as found in yeast (Feser et al., 2010). Although levels of endogenous H3 protein were decreased in aged compared to young adult *glp-1* worms (Ni et al., 2012) a recent study provided evidence that at least a particular H3-variant, HIS-71 increases during aging (Narayan et al., 2016). Of note, changes in the relative levels of individual histone variants have been reported previously to occur during cellular senescence and mammalian aging (Benayoun et al., 2015).

The transcriptional landscape can further be changed by ATP-dependent chromatin remodelers (Clapier et al., 2017) (Table 3). Members of the SWI/SNF complex do at best mildly shorten *C. elegans* wildtype lifespan when inactivated, but are required for DAF-16 dependent processes, such as *daf-2* longevity and dauer formation (Riedel et al., 2013). Conversely, regulation of transcription of *daf-16d/f* by SWI/SNF may contribute to longevity (Bansal et al., 2014), although the particular importance of *daf-16d/f* for lifespan extension in *daf-2* worms (Kwon et al., 2010) has been challenged (Chen et al., 2015). Depletion of *isw-1* (orthologous to the ATPases hSNF2L [NURF-complex] and hSNF2H [CHRAC- and ACF-complexes] (Clapier and Cairns, 2009)), in *daf-2* (Curran et al., 2009) and *cco-1* RNAi animals (Matilainen et al., 2017) shortens their extended lifespan, while *isw-1* overexpression extends wildtype lifespan (Matilainen et al., 2017). On the other hand, loss of *let-418* (Mi2β/CHD4, ATPase of the NuRD complex) also shortens *daf-2* and *glp-1* longevity, while further extending wildtype, *eat-2* and *clk-1* RNAi lifespan (De Vaux et al., 2013). However, effects on wildtype lifespan upon depletion of *isw-1* or *mep-1* (ZNF40), a component of the LET-418 containing MEC-complex, varied depending on the RNAi regimen (Table 3) (Curran et al., 2009; De Vaux et al., 2013; Matilainen et al., 2017; Passannante et al., 2010). Moreover, upon depletion of regulatory subunits of the NURF (Matilainen et al., 2017), CRAC/ACF (Dang et al., 2014), NURD and MEC-complexes (De Vaux et al., 2013), different effects than for depletion of *isw-1/let-418* (Curran et al., 2009; Matilainen et al., 2017; De Vaux et al., 2013) have been reported. Thus, it is possible that these ATPases regulate lifespan through several of their complexes (De Vaux et al., 2013) and that some complexes may play different roles during development and adulthood (Matilainen et al., 2017)

### 4.2. DNA methylation

Directed DNA methylation, at least in mammals, occurs most prominently at the 5-carbon of cytosine (5-methylcytosine, 5-mC) residues in CpG dinucleotides and leads to transcriptional repression (Benayoun et al., 2015; O’Brown and Greer, 2016; Sen et al., 2016). CpG methylation patterns change as humans age and have been proposed as a reliable biomarker of aging (Horvath, 2013). 5-mC is thought to be absent in *C. elegans*, but recent studies detected the presence of 6-methyladenine (6-mA) in worms (Greer et al., 2015) and also in fruit flies (O’Brown and Greer, 2016; Zhang et al., 2015). Subsequent studies provided new evidence for the presence of 6-mA even in mammals and evolutionary conservation of 6-mA regulating methyltransferases and demethylases further supports the concept that 6-mA exerts regulatory functions in multicellular eukaryotes (O’Brown and Greer, 2016). While 6-mA in bacteria serves to distinguish self and foreign DNA (O’Brown and Greer, 2016), it has been implicated into transposon repression and developmental processes in *D. melanogaster* (Zhang et al., 2015) and into the transgenerational regulation of fertility and longevity by the H3K4me2 demethylase SPR-5 (cf. below) in *C. elegans*.

### 4.3. Transgenerational epigenetic inheritance of longevity

Evidence for transgenerational inheritance of longevity was first provided by a study in *C. elegans* which reported that deficiency in H3K4me3 (COMPASS)-complex components (ASH-2/ASH2L, WDR-5/WDR5 or SET-2/SETD1A) extended lifespan not just in mutant animals but also in genetically wildtype progeny from crosses with wildtype worms (Greer et al., 2011). Subsequently, the COMPASS complex was implicated in transgenerational inheritance of increased adult stress resistance when parents, but not progeny, experienced various forms of environmental stress during development (Kishimoto et al., 2017). Similarly, starvation induces transgenerational effects on multiple phenotypes including growth, reproduction and stress resistance (Jobson et al., 2015) and the COMPASS complex, as well as AMPK, ensure reproductive fitness in progeny of starved parents (Demoinet et al., 2017). More recently, another paradigm of transgenerational lifespan regulation was described in *C. elegans* deficient for the H3K4me2 demethylase SPR-5 (Greer et al., 2016). The *spe-5* paradigm differs from the COMPASS paradigm (Greer et al., 2011) in several aspects and appears to transgenerationally regulate a different set of genes (Greer et al., 2016). Of note, transgenerational longevity effects are not observed for wildtype descendants from parents deficient in other chromatin modifiers or established longevity genes such as *utx-1*, *set-9*, *set-15* and *daf-2* (Greer et al., 2011).

## 5. Pharmacologic lifespan extension

Apart from enabling fundamental insights into the biology of aging through genetic studies, C*. elegans* has been proposed to aid in the search for compounds that may promote healthy aging in more complex organism (Lucanic et al., 2017). The most efficient regimen to extend lifespan in model organisms is dietary restriction (Kapahi et al., 2017) and the particular DR-variant of caloric restriction already has been shown to improve health in non-human primates (Mattison et al., 2017). Accordingly, compounds that mimic the effect of DR appear particularly promising in extending healthspan in humans (Calvert et al., 2016; Lucanic et al., 2016). Recent bioinformatics and high throughput experimental screening approaches lead to the identification of candidate CR/DR mimetics in *C. elegans* that now require investigation in higher organisms (Calvert et al., 2016; Lucanic et al., 2016). Additional compounds that recently were shown to increase wildtype *C. elegans* lifespan in candidate testing or small scale screening approaches include small molecules and metabolites such as dimethyl sulfide (Guan et al., 2017), α-ketoacids (Mishur et al., 2016), fructose (Zheng et al., 2017), the d-fructose epimer d-allulose (Shintani et al., 2017), the ω−3 polyunsaturated fatty acid alpha-linolenic acid (ALA) and ALA-derived oxylipin-metabolites (Qi et al., 2017), the proteasome activator 18α-Glycyrrhetinic Acid, a triterpenoid from licorice (Papaevgeniou et al., 2016) and FDA-approved drugs such as rifampicin for tuberculosis (Golegaonkar et al., 2015) and the angiotensin-converting enzyme inhibitor captopril (Kumar et al., 2016) and hydralazine, which are both used to treat hypertension (Dehghan et al., 2017). Many of these compounds apparently act, at least in part, by activating or stabilizing lifespan-regulatory key transcription factors (Table 1), such as *daf-16* (18α-Glycyrrhetinic Acid, rifampicin, captopril), *hlh-30* (selective inhibitors of nuclear export), *hif-1* (α-ketoacids), *nhr-49* (ALA) and *skn-1* (ALA-metabolites, 18 α-Glycyrrhetinic, hydralazine). Moreover, several recent studies described molecular mechanisms of action for *C. elegans* lifespan-extending drugs identified earlier. For example the serotonine and noradrenaline receptor antagonist Mianserin, an antidepressant, has been shown to act by modulating synaptic transmission and cell-non-autonomously inducing oxidative stress response genes in peripheral tissues (Petrascheck et al., 2007; Rangaraju et al., 2015). The nonsteroidal anti-inflammatory drug Aspirin extends *C. elegans* lifespan through mechanisms that overlap with *daf-16-*, *eat-2-*induced DR- and germline signaling (Ayyadevara et al., 2013; Huang et al., 2017; Wan et al., 2013) while the major lipid in bee royal jelly, 10-Hydroxy-2-decenoic acid, acts through the *eat-2*- and TORC1-pathways (Honda et al., 2015, 2011). Intermediate doses of the green tea polyphenol epigallocatechine gallate engage AMPK, *sir-2.1* and *daf-16* for *C. elegans* lifespan extension (Abbas and Wink, 2009; Xiong et al., 2018). Vitamin D exerts beneficial effects on longevity and protein homeostasis via *skn-1*, *ire-1* and *xbp-1* (Mark et al., 2016; Messing et al., 2013). For the antidiabetic drug Metformin, for which first trials have been designed to test their health-promoting effects in humans (Barzilai et al., 2016), multiple mechanisms for *C. elegans* lifespan extension have been reported, including disruption of the folate and methionine cycles in the bacterial food source (Cabreiro et al., 2013), and, in the worm itself, impairment of mitochondrial complex I, TORC1 inhibition and activation of AMPK and SKN-1 (Chen et al., 2017; De Haes et al., 2014; Onken and Driscoll, 2010; Wu et al., 2016b). Importantly, some of these molecular mechanisms appear to be conserved between worms and humans (Chen et al., 2017; Wu et al., 2016b). In summary, these recent reports support the view that *C. elegans* is not just exceptionally useful for uncovering genetic pathways, but also for designing pharmacologic strategies to modulate aging.

## 6. Future perspective

A central question in aging research remains whether extended longevity equates a long and healthy lifespan (Hansen and Kennedy, 2016). Indeed, how genetic and metabolic changes correlate with healthspan has been recently debated. While lifespan extension represents a temporal scaling (Stroustrup et al., 2016), early indications suggested that long-lived *daf-2* animals unexpectedly have lower activity later in life (Bansal et al., 2015; Zhang et al., 2016). However, further studies on healthspan have determined that aging *daf-2* animals are not necessarily unhealthy (Hahm et al., 2015; Podshivalova et al., 2017). One of the important goals in aging research will remain to carefully determine whether lifespan extending interventions maintain a satisfying level of health in the later stages of life.

Multiple studies highlight that reducing the load of aggregating toxic proteins improves fitness and contributes to longevity downstream of many, if not all, longevity pathways. It remains less clear if the stress signaling pathways responsible for clearing aggregates also have broader beneficial effects, including perhaps metabolic changes or alterations in protein synthesis. In addition, organelle remodeling is emerging as a component of cyto-protective mechanisms in cells. For instance, mitochondrial dynamics has recently been linked to longevity (Chaudhari and Kipreos, 2017; Weir et al., 2017). Moving forward, characterizing interactions and identifying biochemical and genetic mechanisms for coordination between tissues and organelles will be key to better understand how cells respond to nutrient signaling and stress to protect the soma.

## Figures and Tables

**Table 1:** Major longevity pathways and longevity-associated transcription factors in *C. elegans.* Other classes of regulators such as micro RNAs and transcriptional coregulators were omitted for simplicity. Green shading indicates that a factor is required for a particular lifespan-extending treatment (RNAi or loss/reduction of function mutation, or dietary restriction regimen) to extend lifespan or to maintain normal lifespan in otherwise wildtype animals. Yellow shading indicates a partial requirement, red shading no requirement, dark green further extension, and white not explicitly tested. sDR: solid DR, lDR: liquid DR. Cf. (Greer and Brunet, 2009) for a more detailed description of these dietary restriction regimens. Note that (Greer and Brunet, 2009) list additional DR-methods not included in this table (Chen et al., 2009; Dillin et al., 2002; Feng et al., 2001; Gerisch et al., 2001; Hansen et al., 2005; Honjoh et al., 2009; Houthoofd et al., 2003; Hsin and Kenyon, 1999; Hsu et al., 2003; Kaeberlein et al., 2006; Kenyon et al., 1993; Lakowski and Hekimi, 1998; Larsen et al., 1995; Lee et al., 2006; Mehta et al., 2009; Morley and Morimoto, 2004; Park et al., 2010; Robida-Stubbs et al., 2012; Senchuk et al., 2018; Seo et al., 2013; Steinbaugh et al., 2015; Steinkraus et al., 2008; Vellai et al., 2003; Zhang et al., 2009; Lee et al., 2010; Lapierre et al., 2013; Panowski et al., 2007; Sheaffer et al., 2008; Tullet et al., 2008; Johnson et al., 2014; Nakamura et al., 2016; Ratnappan et al., 2014; Heestand et al., 2013; Goudeau et al., 2011; Wei and Kenyon, 2016; Lapierre et al., 2011; Van Gilst et al., 2005a; Henderson and Johnson, 2001; Lin et al., 2001; Arum and Johnson, 2007; Walter et al., 2011; Baruah et al., 2014; Chang et al., 2017a; Ventura et al., 2009; Thondamal et al., 2014; Bishop and Guarente, 2007; Greer and Brunet, 2009; Greer et al., 2007; Ching et al., 2010; Burkewitz et al., 2015).

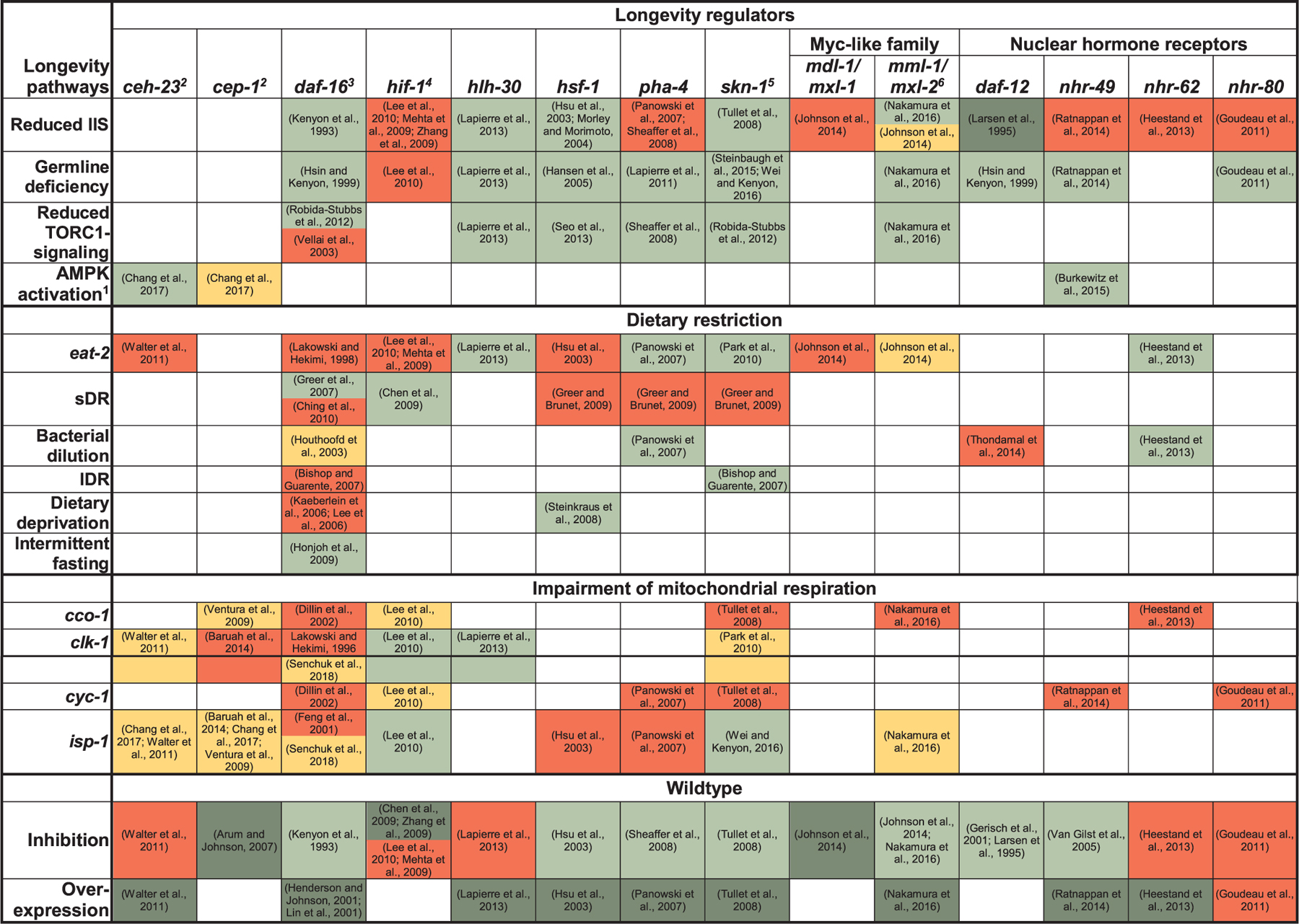

Notes.

1 AMPK activation achieved by transgenic overexpression of a constitutively active *aak-2* (AMPKα) construct; note that *aak-2* is also required for longevity upon sDR and mutation of the TORC1 substrate *rsks-1* (Chen et al., 2013; Greer and Brunet, 2009; Greer et al., 2007), partially upon bacterial dilution and *daf-2, isp-1 or clk-1* mutation (Apfeld et al., 2004; Chen et al., 2013; Curtis et al., 2006; Greer and Brunet, 2009), but not upon *eat-2* mutation or germline deficiency (Curtis et al., 2006; Greer and Brunet, 2009); *aak-2* mutants are shorter-lived than wildtype (Apfeld et al., 2004).

2 *ceh-23* and *cep-1*: these two transcription factors act in a common pathway to modulate lifespan of ETC-compromised worms (Chang et al., 2017a).

3 *daf-16*: sDR-regimens used by (Greer et al., 2007) and (Ching et al., 2010) differed in terms of plate preparation and were initiated at different times of life (day 4 of adulthood vs day 1 of adulthood); isoforms used in overexpression studies in wildtype were *daf-16a1* (Lin et al., 2001) and *a2* (Henderson and Johnson, 2001).

4 *hif-1*: Differences in the observed effects of *hif-1* null mutations on wildtype lifespan may in part be due to different temperature regimens used in the respective studies (Lee et al., 2010)

5 *skn-1*: Effect of overexpression of skn-1 on lifespan was examined using a transgene coding for the SKN-1B/C isoforms (Tullet et al., 2008).

6 *mml-1/mxl-2*: Although (Johnson et al., 2014) and (Nakamura et al., 2016) both used the same mutants [*mml-1(ok8499), mxl-2(tm1516)*], culture conditions differed in terms of the bacterial food source (HT115 vs OP50) and the use of FUDR (400 μM vs no FUDR).

**Table 2:** Methyl marks and their regulators implicated in **C. elegans** lifespan modulation. Mammalian orthologs of regulators are given in parentheses. Effect of the methyl mark on chromatin: A/activating, R/repressive; Change with age (globally): ≈/unchanged, ↓/decreased, ↑/increased; Enzymatic activity: +/methyltransferase forming the respective mark, −/demethylase removing the respective mark; lifespan effect (of knockdown/depletion of the regulator in wildtype worms): ≈/unchanged, ↓/decreased, ↑/increased, tg/transgenerational effect (Greer et al., 2010, 2014, 2016; McColl et al., 2008; Maures et al., 2011; Ni et al., 2012; Towbin et al., 2012; Tian et al., 2016; Merkwirth et al., 2016; Wang et al., 2018; Hamilton et al., 2005; Tian et al., 2016; Maures et al., 2011; Labbadia and Morimoto, 2015; Pu et al., 2015; Jin et al., 2011).

Mark	Effect	Change with age	Regulator (ortholog)	Enzymatic activity	Lifespan effect	Germline dependent^1^	Ref
H3K4 me1/2	A		SET-17 (PRDM7,−11)	+	tg ≈		(Greer et al., 2016; Greer et al., 2014)
			SET-30 (SMYD1–3)		tg ↑		
			LSD-1^2^ (LSD1/KDM1A)	−	↑		(Maures et al., 2011; McColl et al., 2008)
			SPR-5 (LSD1/KDM1A)		tg ↑	No	(Greer et al., 2016)
H3K4 me3	A	≈^3^	SET-2 (SETD1A,B/KMT2F,G)	+	↑	Yes	(Greer et al., 2010)
			RBR-2^4^ (JARID1A,B/KDM5A,B)	−	↓/↑^5^	Yes/no^5^	(Greer et al., 2010; Ni et al., 2012)
H3K9 me2	R		MET-2 (SETDB1/KMT1E)	+	↓		(Tian et al., 2016; Towbin et al., 2012)
			JMJD-1.2^6^ (PHF8)	−	≈		(Merkwirth et al., 2016)
H3K9 me3	R	↓^3^	SET-26 (SETD5, KMT2E)^7^	+	↑/↑^5^	no^5^	(Greer et al., 2010; Hamilton et al., 2005; Ni et al., 2012; Wang et al., 2018)
			SET-25 (EHMT2/KMT1C)	+	≈		(Tian et al., 2016; Towbin et al., 2012)
			JMJD-2^8^ (JMJD2A-D/KDM4A-D)	−	↑^5^/ tg ≈	Yes^5^	(Greer et al., 2016; Greer et al., 2014; Ni et al., 2012)
H3K27 me2	R		JMJD-1.2^6^ (PHF8)	−	≈		(Merkwirth et al., 2016)
H3K27 me3	R	↓^3^	MES-2 (EZH2/KMT6)^9^	+	↑^5^	No^5^	(Ni et al., 2012)
			UTX-1 (UTX/KDM6A)	−	↑/↑^5^	No/no^5^	(Jin et al., 2011; Maures et al., 2011; Ni et al., 2012)
			JMJD-3.1^6^ (JMJD3/KDM6B)	−	≈		(Labbadia and Morimoto, 2015; Merkwirth et al., 2016)
H3K36 me3	A^10^	≈^3, 11, 12^	MET-1 (SETD2/KMT3A)	+	↓	No	(Pu et al., 2015)

Notes.

1 Germline dependence assessed by measuring lifespan of sterile *glp-1(e2144ts)* worms; germline dependency means that deficiency/knockdown of the regulator is not able to modulate lifespan in germline-deficient *glp-1(e2144ts)* worms.

2 Catalytic activity as H3K4me1/2-generating methyltransferase not firmly established (reviewed in (Greer and Shi, 2012)).

3 Experiments to asses global levels of H3K4me3, H3K9me3, H3K27me3 and H3K36me3 in young compared to aged worms were conducted in *glp-1(e2144ts)* worms (Ni et al., 2012); the effect of age on H3K27me3 in *glp-1* animals was confirmed in (Maures et al., 2011).

4 *rbr-2* also displays H3K4me2-demethylase activity, at least *in vitro* (Christensen et al., 2007).

5 Lifespan experiments conducted in the presence of FUDR in (Hamilton et al., 2005; Ni et al., 2012) and in some experiments in (Jin et al., 2011).

6 Reported as H3K9/27me2 (JMJD-1.2) and H3K27me3 (JMJD-3.1) demethylases in *C. elegans*,(Agger et al., 2007; Kleine-Kohlbrecher et al., 2010) but, as discussed in (Merkwirth et al., 2016), the mammalian ortholgs PHF8 and JMJD3 display broader substrate-specificity.

7 The highly similar *set-26* paralog *set-9* was identified as a lifespan regulator in an RNAi-study (Ni et al., 2012), but a recent study using mutants indicated that only *set-26* can modulate lifespan (Wang et al., 2018). SET-9/26 were predicted to be catalytically inactive (Ni et al., 2012) and one study providing *in vitro* evidence that SET-26 mediates H3K9me3, but not methylation of other H3-lysine residues (Greer et al., 2014) is opposed by another study that found no decrease in H3K9me3 upon *set-9/26* inactivation *in vivo*, but suggested that SET-9/26 bind to H3K4me3 (Wang et al., 2018).

8 JMJD-2 also demethylates H3K36me3/2/1 *in vitro* (Greer et al., 2014).

9 EZH2, as part of the Polycomb repressive complex 2 (PRC2), has been reported to regulate all forms of H3K27 methylation (Cao et al., 2002; Ferrari and Pasini, 2013). The study that found a role for *C. elegans* MES-2 regulating H3K27me2/3 levels did not examine H3K27 monomethylation (Bender et al., 2004).

10 Also suppresses cryptic transcription, which is increased in aged (FUDR-treated) worms (Sen et al., 2015).

11 Genome-wide, H3K36me3 patterns do not dramatically change during aging, but gain/loss of H3K36me3 is observed at a subset of genes (Pu et al., 2015).

12 Experiment conducted by (Pu et al., 2015) in germline-deficient *glp-1(e2144ts)* worms.

**Table 3:** Role of ATP-dependent chromatin remodelers in **C. elegans** lifespan regulation. Green shading indicates that a factor is required for a particular lifespan-extending treatment (RNAi or loss/reduction of function mutation or Dietary restriction regimen) to extend lifespan or to maintain normal lifespan in otherwise wildtype animals. Yellow shading indicates a partial requirement, red shading no requirement, dark green further extension, and white not explicitly tested. Function refers to the function of a particular factor within the ATP-dependent chromatin-remodeling complex (c: catalytic, or r: regulatory subunit) (Riedel et al., 2013; Curran et al., 2009; De Vaux et al., 2013; Matilainen et al., 2017; Samuelson et al., 2007; Dang et al., 2014).

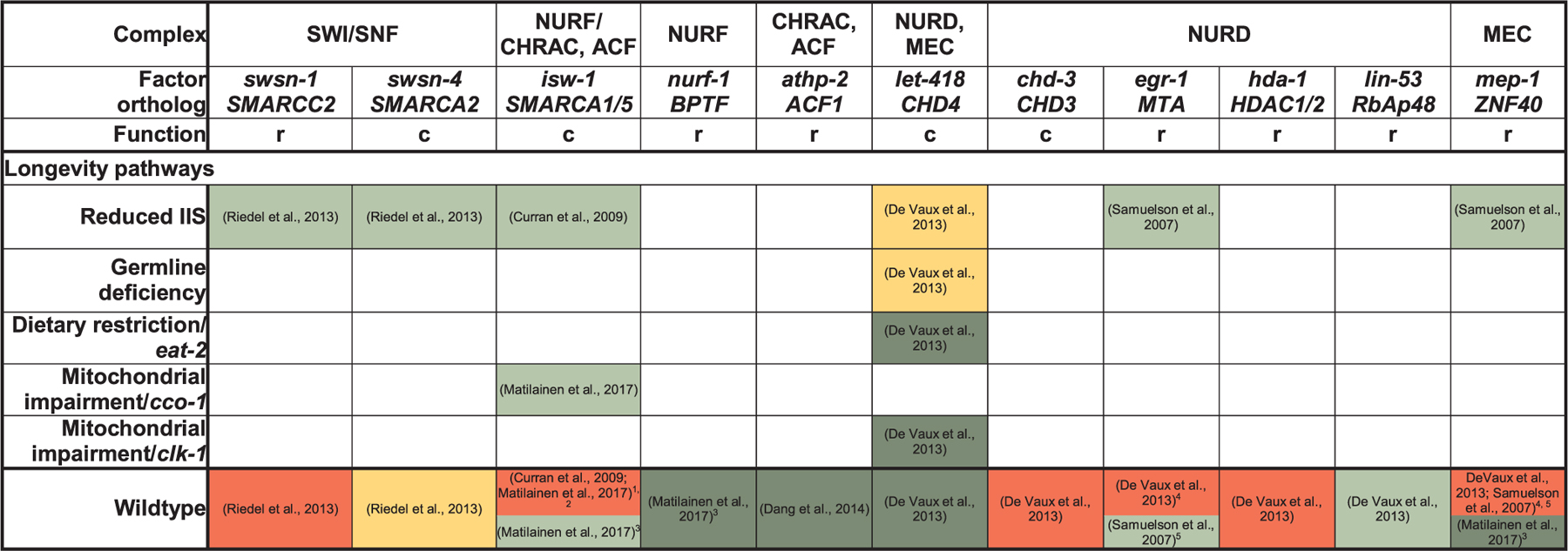

Notes.

1 RNAi was performed only during adulthood by (Curran et al., 2009).

2 The (Matilainen et al., 2017) study used different RNAi regimens and in some cases, also examined mutants; in this case, RNAi was performed from L1.

3 Cf. previous note; different RNAi-regimens were applied in the (Matilainen et al., 2017) study; in this case, RNAi was initiated already in the parental generation starting in L1-L3 and the experimental F1 was kept on RNAi-plates.

4 The (De Vaux et al., 2013) study examined genetic mutations for all genes of interest, with the exception of *egr-1* and *hda-1*, which were knocked down by RNAi starting in L4. Other experimental conditions (lifespans measured at 25 °C, use of FUDR) were the same than in the (Samuelson et al., 2007) study.

5 The (Samuelson et al., 2007) study used RNAi from L4.
